# Neurology and physician-assisted suicide: glossary of definitions and terminology

**DOI:** 10.1007/s10072-025-08064-3

**Published:** 2025-02-26

**Authors:** Alessandra Solari, Nicola Ticozzi, Giancarlo Comi, Gianluigi Mancardi, Leandro Provinciali, Alessandro Padovani, Eugenio Pucci

**Affiliations:** 1 Neuroepidemiology Unit, Fondazione IRCCS Istituto Neurologico Carlo Besta, Milan, Italy; 2 Department of Neuroscience, IRCCS Istituto Auxologico Italiano, 20149 Milan, Italy; 3 Department of Pathophysiology and Transplantation, Università degli Studi di Milano, 20122 Milan, Italy; 4 Dipartimento di Scienze Neuroriabilitative, Casa di Cura Igea, Milan, Italy; 5 Department of Neuroscience, Rehabilitation, Ophthalmology, Genetics, Maternal and Child Health, University of Genoa, Genoa, Italy; 6 Professore Emerito di Neurologia, Università Politecnica delle Marche, Ancona, Italy; 7 Neurology Unit, Department of Clinical and Experimental Sciences, University of Brescia, Brescia, Italy; 8 Department of Continuity of Care and Frailty, Neurology Unit, ASST Spedali Civili Hospital, Brescia, Italy; 9 Neurobiorepository and Laboratory of Advanced Biological Markers, University of Brescia and ASST Spedali Civili Hospital, Brescia, Italy; 10 Brain Health Center, University of Brescia, Brescia, Italy; 11 Azienda Sanitaria Territoriale Fermo, UOC Neurologia, Fermo, Italy; 12 Unit of Neuroepidemiology, Fondazione IRCCS Istituto Neurologico Carlo Besta, via Celoria 11, 20133 Milan, Italy

**Keywords:** Definitions, Glossary, Physician-assisted suicide, Chronic neurological illness, Neuropalliative care/Ethics

## Abstract

The progress of biomedical technologies has expanded the boundaries of care, offering new treatment options and prolonging patients’ survival even in conditions of great suffering and dependence. End-of-life decisions are an important topic in contemporay bioethics and physician-assisted suicide (PAS) is a still controversial topic, with Neurology at the forefront.

This glossary provides a set of definitions to improve clarity and use of a consistent language in the bioethical debate on patients’ autonomy, treatment limitations, palliative care, PAS, euthanasia, and related issues. The objective of the glossary, which is aligned with most recent international consensus documents and with the italian regulatory frameworks, is to clarify and harmonize the practical use of terms.

The glossary, together with the companion paper outlining the position of the Italian Society of Neurology (SIN) regarding PAS, are living documents subject to revision. They can serve as a foundation for a pluralistic discussion in the debate on the ethical issues related to the care of individuals with neurological diseases characterized by poor prognosis in terms of severe disability or limited biological and biographical life expectancies, as well as their families and caregivers. An even more ambitious goal of these documents is to enhance research and inform the development of national regulation in these areas.

**Supplementary Information:**

The online version contains supplementary material available at 10.1007/s10072-025-08064-3.

## Background

Effective treatments are currently available for patients suffering of progressive, severely disabling disorders. These advancements have extended the lives of many patients and, in many cases, have improved patients’ quality of life (QOL). However, there are times when these same treatments may merely prolong the dying process, leading to increased suffering and costs rather than truly improving lives [1]. In high-income countries most patients die in hospitals. Some of these patients receive burdensome supports and technology-intensive care at the end of life (EOL) as a default trajectory [2].

EOL situations remain highly contentious within contemporary bioethics, and Neurology is at the forefront of this debate. Most of the cases faced by the Italian courtrooms and public opinion are neurological, including those of Piergiorgio Welby [3], Eluana Englaro [4], Fabiano Antoniani [5], Federico Carboni [6] (and all those who subsequently requested access to physician-assisted suicide - PAS).

These cases have led to the jurisprudential developments that resulted in the Law 219/2017 [7] and in the Italian Constitutional Court (ICC) Judgments 242/2019 [8] and 135/2024 [9].

The topic of EOL care inevitably intersects with that of palliative care (PC). Neurology, particularly in Italy, is indeed lagging behind, despite Italy having enacted a law on PC in 2010 (Law 38/2010) [10]. Notably, EOL and PC are of interest to young neurologists but remain poorly implemented in educational contexts, not only in Italy [11].

The critical nature of EOL decision-making, coupled with the underlying social, ideological, and political conflicts [12, 13], has made legislative progress extremely difficult. Therefore, although various entities, including the ICC [8], have urged the Italian Parliament to legislate, the process remains stalled. In fact, there are even renewed attempts to amend concepts and data that seemed to have been definitively acquired on a legal level, given their solid clinical underpinnings. A recent example is a bill presented to the Italian Senate [14] that denies that artificial nutrition and hydration are medical treatments, contradicting international guidelines [15–17]. Referring to the same example, the Pontifical Academy for Life has instead supported the validity of considering artificial nutrition and hydration as medical treatment, recognizing ethically legitimate reasons to withdraw it or not initiate it [13].

In the Italian neurological context, documents of the Bioethics and Palliative Care Study Group of the Italian Society of Neurology (SIN) have been published over the last decade [18–20]. Some European Academy of Neurology (EAN) papers also emphasize the role of neurologists in encouraging patients to discuss their wishes about future care, including the limitation of treatments [21–23]. However, there are no specific documents outlining the EAN’s position on EOL.

This glossary provides a set of definitions to improve clarity and use of a consistent language in the bioethical debate on patients’ autonomy, treatment limitations, EOL care, PC, euthanasia, PAS and related topics. The Table lists each term, alternative expressions, and the corresponding Italian term. Considering the heterogeneity of bioethical and legal approaches in the international landscape, the terms defined in the Table as alternatives cannot be regarded as synonyms.

The glossary, together with its companion manuscript [24], are living documents subject to revision. They can serve as a foundation for a pluralistic discussion of the different positions that may arise in the clinical debate on the ethical issues related to the care of individuals with neurological diseases characterized by poor prognosis in terms of severe disability or limited biological and biographical life expectancies, as well as their families and caregivers. An even more ambitious goal of these documents is to facilitate research and inform the development of Italian bioethics and Law relating to these areas. Finally, by referring to the Italian context, they could be valuable aids for international comparison on the topics addressed.

The two manuscripts were developed as follows. In a first meeting (teleconference) the panel agreed on the issues to be addressed in each manuscript. The pertinent literature and documents were retrieved. Three of the authors (AS, LP, EP) drafted each manuscript, that was then circulated to the other panelists for independent revision. In a second meeting, the proposed revisions were discussed and an intermediate version was produced. In the same meeting, the specific positions of the SIN [24] was agreed on via consensus. Further discussion via email exchange was then carried out, and the glossary was reviewed by two experts in law and bioethics. A final version of each manuscript was then produced. The final versions were approved by the Steering Committee of the SIN. An Italian version of the glossary is available as a Supplementary File (Table 1).

**Table 1:** The terms of the glossary, listed in alphabetical order. For each term, alternative expressions and the corresponding Italian term(s) are reported

English term (acronym)	Alternative term	Italian term (acronym)
Advance care planning (ACP)	--	Pianificazione condivisa delle cure (PCC)
Advance directives (ADs)	Advance healthcare directives Advance treatment provisions^1^ Advance care plan	Disposizione anticipate Disposizioni anticipate di trattamento (DAT) Biotestamento^2^ Testamento biologico^2^ Documento di pianificazione condivisa delle cure
Advance treatment provisions^1^	Durable power of attorney	Disposizioni anticipate di trattamento (DAT) Biotestamento^2^ Testamento biologico^2^
Assisted death^2^	Assisted dying^2^ Medical assistance in dying^2^	Morte assistita^2^ Morte medicalmente assistita^2^ Aiuto medico a morire
Care needs	--	Bisogni di cura Bisogni assistenziali
Decision-making capacity	Capacity Competence Mental capacity	Capacità decisionale Capacità di intendere e di volere
Deep continuous palliative sedation	--	Sedazione palliativa profonda continua
Do not resuscitate (DNR) order	Do not attempt resuscitation (DNAR) order	--
End of life (EOL)	--	Fine vita
Euthanasia	--	Eutanasia
Life-support treatment (LST)	Life-sustaining treatment	Trattamenti di sostegno vitale
Living will	--	Disposizioni anticipate di trattamento (DAT)
Non-treatment decisions (NTDs)	Treatment withdrawal Treatment witholding	Sospensione dei trattamenti
Palliative care (PC)	--	Cure palliative
Palliative sedation	--	Sedazione palliativa
Physician-assisted suicide (PAS)	Lawful physician-hastened death Medically-assisted suicide Physician-assisted dying (PAD)	Suicidio medicalmente assistito
Physician orders for life-sustaining treatment (POLST)	--	--
Quality of life (QOL)	--	Qualità della vita
Refractory symptom	--	Sintomo refrattario
Shared decision-making (SDM)	--	Decisioni condivise
Substitute decision-maker	Durable power of attorney Patient advocate Power of attorney Surrogate decision-maker Healthcare agent Healthcare proxy Healthcare surrogate Healthcare attorney in fact	Fiduciario^3^ Incaricato^4^
Total pain	--	Dolore totale
Unfavorable prognosis	--	Prognosi infausta
Voluntary assisted death (VAD)	Hastened death	Morte volontaria assistita Morte volontaria medicalmente assistita

1. “Advance treatment provisions” is the English translation of the Italian DAT

2. Use of this term is not recommended

3. Applies specifically to advance treatment provisions and ACP (Article 5 of the Italian Law 219/2017 [7])

4. Applies specifically to information provision and informed consent (Article 1 of the Italian Law 219/2017 [7])

### Advance care planning (ACP)

“A process that supports adults at any age or stage of health in understanding and sharing their personal values, life goals, and preferences regarding future medical care. The goal of ACP is to help ensure that people receive medical care that is consistent with their values, goals and preferences during serious and chronic illness. For many people, this process may include choosing and preparing another trusted person or persons to make medical decisions in the event the person can no longer make his or her own decisions.” [25].

ACP is regulated by Article 5 of Italian Law 219/2017 [7], In coherence with the SDM model, PCC is a collaborative process between the patient, suffering from a chronic and disabling disease or characterized by unstoppable evolution with a poor prognosis, and their trusted physician. The ACP process includes patient’s reflections on their own values and goals, discussion of possible disease progression, understanding of what the patient can realistically expect in terms of QOL, and of every possible healthcare option, including PC. Other people may be involved in this process upon the patient’s indication, and the appointment of a substitute decision-maker is foreseen but not mandatory. ACP results in the ADs, a set of binding documents to which healthcare professionals working at any facility are required to comply with it if the patient finds himself in the condition of not being able to express his consent/dissent. The AD must be produced in writing or, if the patient’s physical condition does not allow it, by video recording, and kept in the medical record and in the electronic health record. Since a person’s situation changes over time, ACP should be seen as a continuous process that evolves with the person’s changing health states, preferences, and values. This includes adapting to ageing, disability, and the ongoing development of treatment choices that become more specific and relevant to the current context. Thus, ADs resulting from ACP can be updated at any time, at the patient’s request or at the physician’s suggestion. Finally, the ACP updates any AD previously drawn up.

### Advance directives (ADs)

A set of legal documents that primarily include the person’s preferences for future medical treatments (also known as a “living will”) and the appointment of a person who makes decisions on their behalf if they are unable to (substitute decision-maker) [26].

Other types of ADs are DNR order, POLST, and organ donation.

In Western societies, ADs are widely recognized as important means to extend patient autonomy under circumstances of incapacity. However, several differences exist depending on legislation, which in turn reflect different concepts and emphasize various values according to distinct religious, social, and political contexts.

### Advance treatment provisions

This term is the English translation of the “disposizioni anticipate di trattamento” (DAT), and corresponds to the “living will*”* plus, if applicable, the appointment of a substitute decision-maker. Thus, publications that report this term refer specifically to ADs within the Italian context. Advance treatment provisions are regulated by Article 4 of the Law 219/2017 [7]. The provisions of this Law, rooted in the fundamental principles of the Italian Constitution (Articles 2, 13, 32) [27], aim to safeguard the rights to life, health, dignity, and self-determination of individuals across all phases of life, including situations of incapacity to make healthcare decisions. In anticipation of a possible future inability to self-determine and after acquiring adequate medical information on the consequences of one’s choices, this Law provides for the possibility for every adult person with decision-making capacity to express their wishes regarding healthcare treatments, as well as consent or refusal on diagnostic tests and treatments. *“*.The doctor is required to comply with the Advance Treatment Provisions, which can be disregarded, in whole or in part, by the doctor himself, in agreement with the substitute decision-maker, if they are clearly incongruous or do not correspond to the current clinical condition of the patient or if treatments are available that can offer concrete possibilities of improving the patient’s living conditions and which were not foreseeable the time of signing” (Article 4 of the Law 219/17) [7].

Advance treatment provisions can be drawn up at notaries, municipalities, health facilities and, for citizens residing abroad, Italian consulates; they are collected in the National Database of Advance treatment provisions of the Italian Ministry of Health. Advance treatment provisions can be documented not only in writing but also through video recordings, including the use of devices that allow a person with disabilities to communicate.

### Assisted death

Umbrella term referring to both euthanasia and PAS. In accordance with the current position of the American Academy of Neurology (AAN) [28], we suggest avoiding use of this term, as it may be misinterpreted to include the assistance provided by PC during the dying process.

### Care needs

According to the World Health Organization definition of PC [29] there are four dimensions of need: physical, psychological, social, and spiritual. A comprehensive assessment of these needs is the first step for achieving the goals of PC.

Recently, PC has expanded its attention beyond the above-mentioned dimensions, considering information, financial, legal and practical dimensions [30, 31].

### Decision-making capacity

A medical term that refers to whether a patient has the mental capability to: understand relevant information; appreciate their medical situation and its possible consequences; understand the risks, benefits and alternatives of treatment options; and communicate a choice freely and voluntarily based on their own values [32–34].

Capacity is one of the four criteria reported in the ICC Judgment 242/2019 [8] on PAS. Indeed, the ICC refers to the ascertainment of a “patient’s capacity for self-determination and the free and informed nature of the choice they have made”. It is significant that the ICC went beyond the classic view of legal capacity to act (competence), referring instead to the ability to make free and informed decisions, clinically assessed [35]. Although “capacity” and “competence” are often used interchangeably, decision-making competence is a legal term that is determined by the court system, whereas decision-making capacity is a medical term that is determined by the treating physician [33, 34].

All adults are presumed to have capacity, regardless of diagnosis, and healthcare professionals are obligated to promote patients to make as many decisions as their capacity allows. Capacity is assessed intuitively at every medical encounter and is usually readily apparent. However, a structured assessment should be considered if there is reason to question a patient’s capacity [34].

The impact of brain disorders on capacity is a complex issue, requiring case-by-case clinical assessment. Patients can have capacity some of the time, and either have limited capacity or no capacity at other times. Identifying and treating mood disorders in patients requesting VAD is crucial for authentically understanding their desires and ensuring that all clinical interventions have been considered. Clinical depression is common among patients with terminal illnesses and can significantly influence the wish for VAD [36, 37]. However, conditions such as demoralization, hopelessness, and existential distress should also be considered, as they can overlap and may be difficult to interpret clinically [36–38].

This does not mean that depression and related symptoms necessarily impair patient’s capacity. The same can be extended to dementia (considering the spectrum of severity and cognitive impairment) and other mental disorders [39, 40]. At the same time, it is mandatory to maximize patient’s autonomy and ensure access to legal options, even when the primary suffering is due to a mental disorder. Determining that a patient lacks capacity and restricting their autonomy require clear and convincing evidence that the patient’s decision will cause unintended and irreparable harm. If there is uncertainty after conducting a full capacity evaluation, the final judgment should ensure the patient’s choice is respected [33]. In some countries, VAD is available to those suffering from mental disorders (including dementia) also through ADs to avoid discriminatory practices compared to non-mental illnesses and to uphold the principle of justice [39, 41].

### Deep continuous palliative sedation

A type of palliative sedation where the controlled use of sedative drugs is intended to induce unconsciousness continuously until death. Deep continuous palliative sedation aims to alleviate treatment-refractory suffering (i.e., despite the most adequate means having been put in place to control the symptoms) in a patient who has consented to it and who is terminally ill (i.e., probable short-term or imminent death) due to an incurable disease [42, 43].

It is a legitimate and indeed necessary therapeutic procedure from a clinical, ethical, deontological, and legal standpoint, as provided by the Italian Law 219/2017 [7] which states that “the use of deep continuous palliative sedation or the refusal thereof are justified and recorded in the patient’s medical record and electronic medical record.*”*

Although it may involve, as a secondary and unwanted effect, a possible anticipation of the patient’s death, it should no longer be referred to as ‘’indirect active euthanasia’’, a term which, as we reiterate, no longer has meaning and is misleading. There is no evidence-based data on its impact on survival in terms of “biological life”. Without a doubt, it causes the irreversible cessation of “biographical life”.

### “Do not resuscitate” (DNR) order

It is a type of AD, thus a legal document in which a person states not to have cardiopulmonary resuscitation [44], with a specific set of rules.

In Italy there is not a specific ruling for the DNR order. However, provisions on resuscitation and EOL decisions are addressed through a broader legal framework based on informed consent, Advance treatment provisions, and ACP [7].

### End of life (EOL)

The definition of EOL phase varies in the literature. A common definition is one that gives a time frame to the estimated length of life. To this concern, the most commonly cited time frame is a period of fewer than six months of estimated life [45]. However, other literature focuses on the last days or hours of life. A clear definition of time frames relating to EOL can support SDM and ACP, and definition of existing and future healthcare policies and programs. To this concern, it was decided that EOL should refer to patients likely to die within the next 12 months, while the term ‘dying’ to patients in the last days or hours of life [46].

### Euthanasia

The act of intentionally procuring the immediate death by a health professional, by the administration of drugs, of a competent and informed individual who has freely requested it [43].

In some countries around the world euthanasia is legal [24]; the first country to do so was the Netherlands from April 1, 2002. In Italy, euthanasia is a crime punishable under Article 579 of the Penal Code (murder of the consenting party) [47].

The term “passive euthanasia” has often been used and persists in some contexts to describe decisions like treatment withholding, often among health professionals who have instead expressed support in a deceptive manner. This expression is misleading and incorrect, and should not be used at all as euthanasia is an active and voluntary act. The expression of this voluntariness is not necessarily actual, but can be anticipated by means of an AD, as in the case of the Dutch law [24]. If the physician prescribes and/or prepares the drug capable of causing death that is self-administered by the patient, the PAS occurs.

Likewise, the omission of an act that leads to death does not constitute “passive euthanasia”, and can only be traced back to: (a) abstention by the physician, clinically founded and justified, from any unreasonable obstinacy in the administration of treatment and from the use of useless or disproportionate treatments (Article 2 Law 219/2017) [7]; (b) patient’s refusal (current, anticipated by ADs, or reconstructed) (Articles 1, 4, 5 Law 219/2017) [7]. See also non-treatment decisions.

### Life-support treatment (LST)

One of the most authoritative definitions of LST comes from the American Medical Association (AMA): “LST is any treatment that serves to prolong life without reversing the underlying medical condition. LST may include, but is not limited to, mechanical ventilation, renal dialysis, chemotherapy, antibiotics, and artificial nutrition and hydration” [48].

LST is one of the four mandatory criteria for legitimizing PAS in the ICC Judgment 242/2019 [8]. This criterion is unique to Italy, as it is not present in the countries where PAS is legalized, or decriminalized [24].

The phrase “but is not limited to” in the above-mentioned AMA definition highlights the complexity of LST criterion, as emphasized by the Italian Committee for Bioethics (Comitato Nazionale per la Bioetica - CNB) in the Italian context [49]. For instance, if a tetraplegic patient has absolute and complete dependence on a caregiver for the fulfillment of their vital needs, such as feeding, is this considered an LST? That was the case with the ‘Anna case’ [50].

The ICC was asked by a referring judge to widen the criteria for accessing PAS. In the judgement 135/2024 [9] the ICC better defined the meaning of LST, extending them well beyond the previous judgement, to include procedures - such as manual stool evacuation, bladder catheterization or assisted coughing - usually performed by healthcare professionals, but which can also be learned by family members or caregivers, provided that their withdrawal predictably determines the death of the patient in a short period of time.

The ICC also clarified that, for the purposes of accessing PAS, no distinction can be made between the condition of a patient already receiving LSTs, which the patient may request to discontinue, and that of a patient not yet receiving such treatments but now in need of them. Even the patient in the latter condition can legitimately refuse to start LSTs, based on what is outlined in ICC Judgment 242/2019 [8, 9].

### Living will

A legal document that is part of the ADs containing the person’s preferences for medical treatment (or not). This definition is also used (unproperly) as a synonymous for AD.

### Non-treatment decisions

The act of withholding (not starting or increasing) or withdrawing (discontinuing) medical treatment either because of medical futility or at that person’s voluntary and competent request, even as documented in ADs. Treatment withdrawal or withholding is therefore not comparable to euthanasia.

In Italy, the Law 219/2017 [7] states that “no treatment may be started or continued without the free and informed consent of the person concerned, except in cases expressly provided for by law” and recognises the right of any competent person to refuse or interrupt any treatment, even if it is essential for one’s survival, including artificial nutrition and hydration. In fact, the physician is “required to respect the patient’s expressed will to refuse medical treatment or to renounce it and, as a result, is exempt from civil or criminal liability” (Article 1 paragraph 6). The request for suspension of medical treatment may be associated with the request for PC (including deep continuous palliative sedation) to alleviate the patient’s suffering.

### Palliative care (PC)

The Italian Law 38/2010 defines PC: “the set of therapeutic, diagnostic, and supportive interventions, directed both at the patient and their family, aimed at the active and total care of patients whose underlying disease, characterized by an unstoppable progression and a poor prognosis, no longer responds to specific treatments.” [51]. This definition emphasizes that PC should be offered to any patients experiencing complex symptoms and needs, both during treatments intended to slow down the disease (early and simultaneous PC), and when such treatments are no longer effective (EOL PC). PC aims to prevent or control/alleviate physical, as well as psychological, social, and spiritual suffering (see total pain) induced by the underlying condition. In addition, it seeks to enhance patient self-determination, social inclusion, and communication with their loved ones, while ensuring continuity of care in any setting (from home to hospital), in order to improve the patient’s QOL, also paying attention to patients’ loved ones, until the EOL and, for the loved ones, even during bereavement [52]. Integration of PC into intensive care units has been shown to result in shorter hospital lengths of stay, an increase in ADs, and a decrease in the use of nonbeneficial life-prolonging treatments [2, 53].

The benefits of PC in enhancing patient outcomes have become increasingly evident, particularly regarding symptom control, overall QoL, and care satisfaction of both patients and families, including those with neurological conditions [21–23]. Nevertheless, integrating PC earlier in the disease trajectory remains a challenge. Furthermore, there is frequently a lack of clarity about the terminology used for implementing PC and who is responsible for delivering these services.

There are different models for delivering PC. A model we aim to promote is the dynamic approach to early and simultaneous PC, which integrates PC provided by neurologists (“general PC” or “primary PC”) and “specialist PC”. The latter involves an interprofessional and interdisciplinary team, tailored to the changing needs of the patient and their loved ones [21, 54].

In a 1996 report, the AAN Ethics and Humanities Subcommittee declared that providing primary PC is the responsibility of all neurologists, and this position remains unchanged in the 2022 position paper [55, 56].

Since the publishing of the 1996 report, the AAN has developed a true field of neuropalliative care, framing it as a subspecialty within neurology and PC and defining it as an “approach as palliative care that focuses on the specific needs of patients with neurologic illness and their families.” [57]. A similar recognition has been provided by the EAN, which has established an EAN Scientific Panel on PC (https://www.ean.org/home/organisation/scientific-panels/palliative-care) and promoted a consensus review [21].

PC is defined as opposing both the hastening and prolongation of the terminal phase of life. Indeed, almost all definitions of PC over the years have confirmed the assertion that “PC views death as a natural event and neither accelerates nor delays death” [58]. Even in countries where euthanasia and/or PAS are legal (e.g., Netherlands, Belgium, Luxembourg, Canada) [24], the debate within the field of PC has always been very active, and the scientific societies of PC have expressed opposition to these practices, although there is a minority position among health professionals who have instead expressed support for these practices [59].

### Palliative sedation

*“*The monitored use of medications intended to induce a state of decreased or absent awareness (unconsciousness) in order to relieve the burden of otherwise intractable suffering in a manner that is ethically acceptable to the patient, family and healthcare providers*”* [60]. There is no solid evidence-based data that palliative sedation hastens death; and in some cases, it may, on the contrary, briefly prolong the patient’s remaining life even within the context of imminent terminal illness [61].

In accordance with the definitions suggested by the Italian CNB [42], palliative sedation can be administered in several ways. Based on the level of consciousness, it can be moderate/surface (consciousness is not completely abolished) or deep (consciousness is completely abolished). Based on duration, it can be: temporary (for a limited period); intermittent (administered alternately based on changing circumstances); or continuous (extended until the patient’s death; i.e., deep continuous palliative sedation).

### Physician-assisted suicide (PAS)

“A physician intentionally helping a person to terminate his or her life by providing drugs for self-administration, at that person’s voluntary and competent request” [43].

In Italy, assisted suicide is a crime punishable by up to five to twelve years’ imprisonment, according to Article 580 of the Criminal Code (instigation or assistance to suicide) [47].

ICC Judgment 242/2019 [8] has placed an exception to punishability of assisted suicide. In fact, the ICC stated the illegitimacy of Article 580 “in so far as it does not exclude the punishment of those who (.) facilitates the fulfilment of the autonomously and freely formed intent to commit suicide of a person fully capable of making free and informed decisions kept alive by life-support treatments and suffering from an incurable illness which is a source of physical or psychological suffering that he or she considers intolerable, provided that these conditions and the method of implementation have been verified by a public national health service facility after consulting the territorially competent ethics committee.” [8]. For the Judgement to apply it is required that the person’s will has been clearly and unequivocally expressed, compatibly with what is permitted by their conditions. It is also required that the person has been adequately informed of the PAS and of possible alternative solutions, in particular about access to PC and, where appropriate, palliative sedation.

In the absence of specific legislation, this Judgment lays the foundation for the current access to PAS, which is far from being clarified, with procedures still inconsistent across the national territory. For the requirements established by the ICC, see also the following terms of this glossary: decision-making capacity, life-support treatment, and unfavourable prognosis.

### Physician orders for life-sustaining treatment (POLST)

A POLST is a type of ADs for people who have been diagnosed with a serious illness. It is a binding document completed by the patient’s referral physician which specifies the life-sustaining treatments that should be given to the patient, under circumstances of incapacity [62]. In Italy, the concept of POLST is not formally recognized.

### Quality of life (QOL)

“The perception that individuals have of their position in life in the context of the culture and value systems in which they live and in relation to their goals, expectations, standards, and concerns” [63]. QOL is a multi-dimensional concept: a minimum of three inter-related dimension (physical, psychological, and social) contribute to the individual’s overall QOL [64]. In addition to multi-dimensionality, subjectivity is a fundamental feature of QOL. The best assessor of this construct is the individual, using self-administered instruments. The use of standardized QoL instruments has increased exponentially over time in clinical research, and both generic and condition-specific QOL instruments have been produced and validated across languages and cultures. In neurology, QOL assessment is crucial for understanding the full impact of diseases and their possible treatment on patients’ lives [65]. This is the case also for PC, where the QOL of the patient’s significant others is also a key outcome [52].

### Refractory symptom

“A symptom for which all possible treatment has failed, or it is estimated that no methods are available for palliation within the time frame and the risk–benefit ratio that the patient can tolerate […] that does not compromise consciousness” [60]. In other words, refractory symptoms are present in situations where the best possible management with conventional treatments have failed or cannot be utilized, such as when side effects are unacceptable to the patient, or the effects take too long to relieve the symptom within an appropriate timeframe [66].

Patients with severe neurological disability often experience a range of persistent distressing symptoms. An incomplete list of symptoms that may become resistant to standard therapies and are categorized as refractory includes, but is not limited to breathlessness, pain, delirium and agitation, nausea, epileptic seizures, and existential distress (including death-related distress). They are not limited to physical symptoms; therefore, a multidimensional approach is needed to fully assess and manage the patient’s condition [67].

When therapeutic approaches have failed and symptoms are deemed refractory to treatment, particularly near EOL, palliative sedation becomes a prominent consideration [60].

### Shared decision-making (SDM)

An evidence- and ethically-based model in which informed decisions are collaboratively made by physicians and patients based on the best available evidence and the patient’s values and preferences [68–70]. SDM is especially important in cases of preference-sensitive decisions that typically occur in chronic medical conditions characterized by variable prognoses and the limited effectiveness of disease-modifying treatments. This is the case for most neurological disorders where such decisions are made from diagnostic workup to the EOL phase [71]. SDM and ACP enhance patient autonomy by engaging patients, physicians and when applicable patients’ significant others in the decision-making process. SDM and ACP are best practice models and part of the medical curricula in many countries [72].

### Substitute decision-maker

A person appointed to ensure compliance with the patient’s choices in the event the patient is no longer able to do so, either permanently or temporarily. It is not mandatory to identify a substitute decision-maker, and in most Western countries, there is a specific AD document for this.

As from the Italian Law 219/2017 [7], it is up to the person to appoint or not appoint a substitute decision-maker, for both advance treatment provisions and ACP.

Notably, the Law 219/2017 uses the specific term of trustee (in italian “fiduciario”) (Table) for substitute decision maker in advance treatment provisions and ACP. In both procedures, multiple substitute decision makers may be appointed and listed in order of preference, with each required to sign the document and provide their contact information.

As far as information provision and informed consent are concerned (Article 1, Law 219/17) [7], the term for substitute decision maker is appointee (in Italian “incaricato”) (Table). Specifically, the patient “may refuse in whole or in part to receive information or may designate one or more family members or a trusted person to receive it and to give consent on the patient’s behalf. The refusal or renunciation of information and any designation of a representative are registered in the medical record and in the electronic medical record” [7].

### Total pain

This term was introduced by Cicely Saunders, the founder of the modern hospice movement, and it encompasses the physical, emotional, social, and spiritual dimensions of distress [73].

### Unfavorable prognosis

In the absence of a description of this expression within the framework of the Italian Law 38/2010 [51], unfavorable prognosis refers to medical conditions where the possibility of recovery is absent or extremely limited, accompanied by a significant decline in the patient’s QOL, and/or by a reduced life expectancy. Therefore, this definition should not be limited to diseases that lead to death but should also extend to situations where the clinical condition has a severe existential impact, requiring complex symptom management that impact the patient’s QOL.

### Voluntary assisted death (VAD)

It is defined by the active and intentional choice of a person to end their life in the context of terminal illness and/or severe suffering. This term encompasses both PAS and euthanasia.

Requests for VAD by patients with neurological diseases can stem from a complex mix of psychological, physical (e.g., pain, fatigue), social, and spiritual factors. These requests often reflect overall suffering and a loss of autonomy and dignity due to an inability to live according to individual values and preferences [74]. Patients with advanced diseases may feel a loss of control over their lives [75], perceiving their situation as intolerable and experiencing a sense of unworthiness. They may feel like a burden, both financially and emotionally, to their families, and social isolation can heighten the desire to die. Clinical depression, common among patients with terminal illnesses, can also influence the wish for VAD (see decision-making capacity).

EAN guidelines suggest encouraging patients to discuss their wishes regarding VAD [22, 23, 76]. It is also important to note that PC, even at its highest quality, cannot prevent patients from requesting VAD [43].

## Electronic supplementary material

Below is the link to the electronic supplementary material.

Supplementary Material 1
