# Supplementary material for: Neurology and physician-assisted suicide: glossary of definitions and terminology
Source: Neurol Sci. 2025 Feb 26;46(6):2381–92. doi: 10.1007/s10072-025-08064-3 (PMC12084260; doi:10.1007/s10072-025-08064-3)
Supplement: Supplementary file 1 — Supplementary Material 1 [file 10072_2025_8064_MOESM1_ESM.docx]

**SUPPLEMENTARY INFORMATION**

Translation into Italian of the article 'Neurology and Physician-Assisted Suicide: Glossary of Definitions and Terminology'. To ensure consistency with the text and references of the original English article, the order of the terms has been preserved.

Traduzione in italiano dell’articolo “Neurology and Physician-Assisted Suicide: Glossary of Definitions and Terminology”. Per mantenere la corrispondenza con il testo e la bibliografia dell'articolo originale in inglese, è stato preservato l'ordine di elencazione dei termini di quest'ultimo.

**NEUROLOGIA E SUICIDIO MEDICALMENTE ASSISTITO: GLOSSARIO**

**BACKGROUND**

Attualmente sono disponibili trattamenti efficaci per i pazienti che soffrono di patologie progressive e gravemente invalidanti. Questi progressi hanno prolungato la sopravvivenza dei pazienti e, in molti casi, ne hanno migliorato la qualità della vita (QdV). Tuttavia, ci sono situazioni in cui gli stessi trattamenti possono limitarsi a prolungare il processo di morte, aumentando la sofferenza e i costi senza migliorare realmente la vita [1]. Nei paesi ad alto reddito, la maggior parte dei pazienti muore in ospedale. Alcuni di questi ricevono trattamenti onerosi e cure intensive basate su tecnologie avanzate alla fine della vita [2].

Le situazioni di fine vita rimangono fortemente controverse all'interno della bioetica contemporanea, e la neurologia è in prima linea in questo dibattito. La maggior parte dei casi esaminati dai tribunali italiani e dall’opinione pubblica riguarda patologie neurologiche, come dimostrano i casi di Piergiorgio Welby [3], Eluana Englaro [4], Fabiano Antoniani [5], Federico Carboni [6] (e tutti coloro che successivamente hanno richiesto l’accesso al suicidio medicalmente assistito - SMA). Questi casi hanno portato agli sviluppi giurisprudenziali che hanno dato origine alla Legge 219/2017 [7] e alle sentenze della Corte Costituzionale (CC) 242/2019 [8] e 135/2024 [9].

Il tema della cura nel fine vita si intreccia inevitabilmente con quello delle cure palliative (CP). La neurologia, in particolare in Italia, è in ritardo rispetto ad altre discipline, nonostante l’Italia abbia promulgato una legge sulle CP nel 2010 (Legge 38/2010) [10]. E’ da segnalare che le cure di fine vita e le CP interessano i giovani neurologi ma rimangono scarsamente implementate nei contesti formativi, non solo in Italia [11].

La complessità delle decisioni di fine vita, unita ai conflitti sociali, ideologici e politici sottostanti [12, 13], rende estremamente difficile il progresso legislativo. Pertanto, sebbene vari soggetti, inclusa la CC [8], abbiano sollecitato il Parlamento italiano a legiferare, il processo è ancora in stallo. In realtà, ci sono persino tentativi di modificare concetti e dati che sembravano essere stati definitivamente acquisiti a livello giuridico, data la loro solida base clinica. Un recente esempio è un disegno di legge presentato al Senato Italiano [14] che nega che nutrizione e idratazione artificiali costituiscano trattamenti medici, contraddicendo le linee guida internazionali [15-17]. In riferimento allo stesso esempio, la Pontificia Accademia per la Vita ha invece sostenuto la validità di considerare nutrizione e idratazione artificiali come trattamenti medici, riconoscendo l’esistenza di ragioni eticamente legittime alla base del rifiuto a proseguire o iniziare questi trattamenti [13].

Nel contesto neurologico italiano, negli ultimi dieci anni sono stati pubblicati documenti del Gruppo di Studio di Bioetica e Cure Palliative della Società Italiana di Neurologia (SIN) [18-20]. Alcuni articoli dell'*European Academy of Neurology* (*EAN*) sottolineano inoltre il ruolo dei neurologi nell'incoraggiare i pazienti a discutere dei loro desideri riguardo alla futura assistenza, compresa la limitazione dei trattamenti [21-23]. Tuttavia, non esistono documenti specifici che delineino la posizione dell'*EAN* sul fine vita.

Questo glossario fornisce un insieme di definizioni per fare chiarezza e favorire l'uso di un linguaggio coerente nel dibattito bioetico su temi quali autonomia dei pazienti, limitazioni dei trattamenti, cure di fine vita, CP, eutanasia, SMA e argomenti correlati. La Tabella elenca ciascun termine, le espressioni alternative e il termine italiano corrispondente. Considerando l'eterogeneità degli approcci bioetici e giuridici a livello internazionale, i termini definiti nella Tabella come alternativi non possono essere considerati sinonimi.

Il glossario, come del resto il *position paper* di cui è completamento [24], è un documento in evoluzione e soggetto a revisione. Entrambe le pubblicazioni serviranno come base per una discussione pluralistica delle diverse posizioni che possono sorgere nel dibattito clinico sulle questioni etiche relative alla cura di individui affetti da malattie neurologiche, caratterizzate da una prognosi infausta in termini di grave disabilità o di aspettativa di vita biologica e/o biografica limitata, nonché delle loro famiglie e *caregiver*. Un obiettivo ancora più ambizioso di questi documenti è facilitare la ricerca e informare lo sviluppo della bioetica e della legge italiana in questi ambiti. Infine, facendo riferimento al contesto italiano, questi documenti potrebbero essere utili per un confronto internazionale sui temi trattati.

I due manoscritti sono stati sviluppati come segue. In un primo incontro (teleconferenza) il *panel* ha concordato le questioni da trattare in ciascun manoscritto. La letteratura e i documenti pertinenti sono stati recuperati. Tre degli autori (AS, LP, EP) hanno redatto ciascun manoscritto, che è stato poi inviato agli altri membri del gruppo per una revisione indipendente. In un secondo incontro, sono state discusse le revisioni proposte ed è stata prodotta una versione intermedia di ciascun manoscritto. Nello stesso incontro, i punti specifici che esprimono la posizione della SIN sono stati declinati tramite consenso [24]. È seguito un ulteriore confronto tramite scambio di email, e il glossario è stato rivisto da due esperti in diritto e bioetica. È stata quindi prodotta una versione finale di ciascun manoscritto. Le versioni finali sono state approvate dal Comitato Direttivo della SIN.

**Tabella.** I termini del glossario riportati in ordine alfabetico, le espressioni alternative (non necessariamente sinonimi), e i termini italiani corrispondenti.

| **Termini inglesi (acronimi)** | **Termini inglesi alternativi** | **Termini italiani (acronimi)** |
| --- | --- | --- |
|  |  |  |
| *Advance care planning (ACP)* | -- | Pianificazione condivisa delle cure (PCC) |
| *Advance directives* | *Advance healthcare directives*  *Advance treatment provisions^1^*  *Advance care plan* | Disposizioni anticipate (DA)  Disposizioni anticipate di trattamento (DAT)  Biotestamento^2^  Testamento biologico^2^  Documento di pianificazione condivisa delle cure |
| *Advance treatment provisions*^1^ | *Durable power of attorney* | Disposizioni anticipate di trattamento (DAT)  Biotestamento^2^  Testamento biologico^2^ |
| *Assisted death*^2^ | *Assisted dying*^2^  *Medical assistance in dying*^2^ | Morte assistita^2^  Morte medicalmente assistita^2^  Aiuto medico a morire |
| *Care needs* | -- | Bisogni di cura  Bisogni assistenziali |
| *Decision-making capacity* | *Capacity*  Competence  Mental capacity | Capacità decisionale  Capacità di intendere e di volere |
| *Deep continuous palliative sedation* | -- | Sedazione palliativa profonda continua |
| *Do not resuscitate (DNR) order* | *Do not attempt resuscitation (DNAR) order* | -- |
| *End of life* | -- | Fine vita |
| *Euthanasia* | -- | Eutanasia |
| *Life-support treatment* | *Life-sustaining treatment* | Trattamenti di sostegno vitale (TSV) |
| *Living will* | -- | Disposizioni anticipate di trattamento (DAT) |
| *Non-treatment decisions* | *Treatment withdrawal*  *Treatment witholding* | Sospensione dei trattamenti |
| *Palliative care* | -- | Cure palliative (CP) |
| *Palliative sedation* | -- | Sedazione palliativa |
| *Physician-assisted suicide* | *Lawful physician-hastened death*  *Medically-assisted suicide*  *Physician-assisted dying (PAD)* | Suicidio medicalmente assistito (SMA) |
| *Physician orders for life-sustaining treatment (POLST)* | -- | -- |
| *Quality of life* | -- | Qualità della vita (QdV) |
| *Refractory symptom* | -- | Sintomo refrattario |
| *Shared decision-making (SDM)* | -- | Decisioni condivise |
| *Substitute decision-maker* | *Durable power of attorney*  *Patient advocate*  *Power of attorney*  *Surrogate decision-maker*  *Healthcare agent*  *Healthcare proxy*  *Healthcare surrogate*  *Healthcare attorney in fact* | Fiduciario^3^  Incaricato^4^ |
| *Total pain* | -- | Dolore totale |
| *Unfavorable prognosis* | -- | Prognosi infausta |
| *Voluntary assisted death* | *Hastened death* | Morte volontaria assistita (MVA)  Morte volontaria medicalmente assistita |

1. "*Advance treatment provisions*" è la traduzione in inglese di DAT.
2. L'uso di questo termine non è raccomandato.
3. Si applica specificamente alle DAT e alla PCC (articolo 5 della Legge Italiana 219/2017 [7]).
4. Si applica specificamente alla trasmissione di informazioni e al consenso informato (articolo 1 della Legge Italiana 219/2017 [7]).

***Advance Care Planning* (*ACP*)**

"Un processo che supporta gli adulti di qualsiasi età o stato di salute nella comprensione e condivisione dei propri valori personali, obiettivi di vita e preferenze riguardo all'assistenza medica futura. L'obiettivo dell'*ACP* è garantire che i pazienti con malattie croniche e gravi ricevano cure mediche coerenti con i loro valori, obiettivi e preferenze. Per molti pazienti, questo processo può includere l’individuazione e la preparazione di una o più persone di fiducia a prendere decisioni mediche nell’eventualità che lo stesso paziente si trovasse nella condizione di non essere in grado di decidere autonomamente" [25].

L'*ACP* è regolato in Italia dall'articolo 5 della Legge 219/2017 [7]. In coerenza con il modello delle decisioni condivise (*shared decision making*), si può realizzare la Pianificazione Condivisa delle Cure (PCC) che è un processo collaborativo tra il paziente, affetto da una patologia cronica e invalidante o caratterizzata da un'evoluzione inarrestabile con prognosi infausta, e il medico di fiducia. Il documento relativo alla PCC deve essere prodotto per iscritto o, se le condizioni fisiche del paziente non lo consentono, mediante videoregistrazione e conservato nella cartella clinica e nel fascicolo sanitario elettronico. Il processo di PCC include la riflessione del paziente sui propri valori e obiettivi, la discussione sull'evoluzione possibile della malattia, la comprensione di ciò che il paziente può realisticamente aspettarsi in termini di QdV e di ogni possibile opzione di assistenza sanitaria, incluse le CP. Altre persone possono essere coinvolte in questo processo su indicazione del paziente, ed è prevista la nomina di un fiduciario, anche se non è obbligatoria. L*'ACP* porta alla redazione delle Disposizioni Anticipate (DA), documenti vincolanti cui i professionisti sanitari che operano in qualsiasi struttura sono tenuti a conformarsi nel caso in cui il paziente si trovi nella condizione di non poter esprimere il proprio consenso/dissenso. Poiché la situazione di una persona cambia nel tempo, l'*ACP* dovrebbe essere considerato come un processo continuo che evolve con i cambiamenti dello stato di salute, delle preferenze e dei valori della persona. Questo include l'adattamento all'invecchiamento, alla disabilità e allo sviluppo continuo delle opzioni terapeutiche che diventano più specifiche e rilevanti per il contesto attuale. Pertanto, le DA risultanti dall'*ACP* possono essere aggiornate in qualsiasi momento, su richiesta del paziente o su suggerimento del medico. Infine, l'*ACP* aggiorna eventuali DA redatte in precedenza.

**Disposizioni Anticipate (DA)**

Un insieme di documenti legali che includono principalmente le preferenze di una persona per i trattamenti medici futuri (noto anche come “testamento biologico”) e la nomina di una persona che prenda decisioni per suo conto qualora non fosse più in grado di farlo (rappresentante per le decisioni sanitarie) [26].

Altri tipi di DA includono *DNR*, *POLST* e la donazione di organi. Nelle società occidentali, le DA sono ampiamente riconosciute come strumenti importanti per estendere l'autonomia del paziente in circostanze di incapacità. Tuttavia, esistono differenze significative a seconda della legislazione, che riflettono concetti distinti e valori diversi in base ai contesti religiosi, sociali e politici.

**Disposizioni Anticipate di Trattamento (DAT)**

Questo termine si riferisce specificamente al contesto italiano.

Le DAT sono regolate dall'articolo 4 della Legge 219/2017 [7]. Le misure di questa Legge, radicate nei principi fondamentali della Costituzione Italiana (articoli 2, 13, 32) [27], mirano a tutelare i diritti alla vita, alla salute, alla dignità e all’autodeterminazione degli individui in tutte le fasi della vita, incluse le situazioni di incapacità di prendere decisioni sanitarie. In previsione di una possibile futura incapacità di autodeterminarsi e dopo aver acquisito informazioni mediche adeguate sulle conseguenze delle proprie scelte, questa Legge prevede la possibilità, per ogni persona maggiorenne con capacità decisionale, di esprimere le proprie scelte riguardo ai trattamenti sanitari, nonché il consenso o il rifiuto a esami diagnostici e cure. “…Il medico è tenuto a rispettare le DAT, le quali possono essere disattese, in tutto o in parte, dal medico stesso, in accordo con il fiduciario, qualora esse appaiano palesemente incongrue o non corrispondenti alla condizione clinica attuale del paziente ovvero sussistano terapie non prevedibili all’atto della sottoscrizione, capaci di offrire concrete possibilità di miglioramento delle condizioni di vita” (articolo 4 della Legge 219/17) [7].

Le DAT possono essere redatte presso notai, comuni, strutture sanitarie e, per i cittadini residenti all’estero, presso i consolati italiani; esse sono raccolte nella Banca dati delle DAT del Ministero della Salute italiano. Le DAT possono essere documentate non solo in forma scritta, ma anche tramite videoregistrazione, incluso l’uso di dispositivi che consentono a persone con disabilità di comunicare.

**Morte assistita**

Termine ombrello che si riferisce sia all'eutanasia sia al SMA. In accordo con la posizione attuale dell'*American Academy of Neurology* [28], suggeriamo di evitare l'uso di questo termine, poiché potrebbe essere frainteso includendo l'assistenza fornita dalle CP durante il processo del morire.

**Bisogni di cura**

Secondo la definizione dell'Organizzazione Mondiale della Sanità sulle CP [29], ci sono quattro dimensioni del bisogno: fisica, psicologica, sociale e spirituale. Una valutazione completa di questi bisogni è il primo passo per raggiungere gli obiettivi delle CP.

Recentemente, le CP hanno ampliato la loro attenzione oltre le dimensioni sopra menzionate, considerando anche le dimensioni informative, finanziarie, legali e pratiche [30, 31].

**Capacità decisionale**

Termine medico che si riferisce alla capacità mentale di un paziente di: comprendere informazioni rilevanti; apprezzare la propria situazione medica e le sue possibili conseguenze; comprendere i rischi, i benefici e le alternative delle opzioni di trattamento; e comunicare una scelta liberamente e volontariamente basata sui propri valori [32-34].

La capacità di prendere decisioni è uno dei quattro criteri riportati nella Sentenza della CC 242/2019 [8] sull’assistenza al suicidio. Infatti, la CC si riferisce all'accertamento della "capacità di autodeterminazione del paziente e il carattere libero e informato della scelta espressa". È significativo che la CC sia andata oltre la visione classica della capacità in termini giuridici, riferendosi invece alla capacità di prendere decisioni libere e informate, valutate clinicamente [35].

Si presume che tutti gli adulti abbiano capacità decisionale, indipendentemente dalla diagnosi, e i professionisti sanitari hanno il dovere di promuovere il coinvolgimento dei pazienti nel prendere quante più decisioni possibile, in base alla loro capacità. La capacità viene valutata in modo intuitivo ad ogni incontro medico ed è di solito facilmente apparente. Tuttavia, dovrebbe essere considerata una valutazione strutturata se ci sono motivi per mettere in discussione la capacità di un paziente [34].

L'impatto dei disturbi cerebrali sulla capacità è una questione complessa, che richiede una valutazione clinica caso per caso. I pazienti possono avere capacità in alcuni momenti e avere capacità limitata o assente in altri. Identificare e trattare i disturbi dell'umore nei pazienti che richiedono la MVA è cruciale per comprendere autenticamente i loro desideri e garantire che tutti gli interventi clinici siano stati considerati. La depressione è comune tra i pazienti con malattie terminali e può influenzare significativamente il desiderio di MVA [36, 37]. Inoltre, condizioni come la demoralizzazione, la disperazione e il disagio esistenziale devono essere considerate, poiché possono sovrapporsi e potrebbero essere difficili da interpretare clinicamente [36, 38]. Ciò non significa che la depressione e i sintomi correlati compromettano necessariamente la capacità del paziente. Lo stesso può essere esteso alla demenza (considerando lo spettro di gravità e il tipo di compromissione cognitiva) e ad altri disturbi mentali [39, 40]. Allo stesso tempo, è obbligatorio massimizzare l'autonomia del paziente e garantire l'accesso a opzioni legali, anche quando la sofferenza primaria è dovuta a un disturbo mentale. Giudicare che un paziente non abbia capacità e limitare la sua autonomia richiede prove chiare e convincenti che la decisione del paziente causerà danni imprevisti e irreparabili. Se c'è incertezza dopo aver condotto una valutazione completa della capacità, il giudizio finale dovrebbe propendere per l’autonomia del paziente [33]. In alcuni paesi, la MVA è disponibile per coloro che soffrono di disturbi mentali (inclusa la demenza) anche attraverso le DA, per evitare pratiche discriminatorie rispetto alle malattie non mentali e sostenere il principio di giustizia [39, 41].

**Sedazione palliativa continua profonda**

Un tipo di sedazione palliativa in cui l'uso controllato di farmaci sedativi è destinato a indurre un'incoscienza continua fino alla morte. La sedazione palliativa continua profonda mira ad alleviare la sofferenza refrattaria al trattamento (cioè, nonostante siano stati messi in atto i mezzi più adeguati per controllare i sintomi) in un paziente che ha acconsentito e che è in fase terminale (cioè, con una probabile morte a breve termine o imminente) a causa di una malattia incurabile [42, 43].

E’ una procedura terapeutica legittima e, in effetti, necessaria dal punto di vista clinico, etico, deontologico e legale, come previsto dalla Legge italiana 219/2017 [7], che afferma che “il ricorso alla sedazione palliativa profonda continua o il rifiuto della stessa sono motivati e sono annotati nella cartella clinica e nel fascicolo sanitario elettronico”.

Sebbene possa comportare, come effetto secondario e indesiderato, una possibile anticipazione della morte del paziente, essa non dovrebbe più essere definita "eutanasia attiva indiretta", un termine che, come ribadiamo, non ha più significato ed è fuorviante. Non ci sono dati basati su evidenze riguardo al suo impatto sulla sopravvivenza in termini di "vita biologica". Senza dubbio, essa causa la cessazione irreversibile della "vita biografica".

**“*Do not resuscitate*" (*DNR*)**

Si tratta di un tipo di DA, quindi di un documento legale in cui una persona dichiara di non voler ricevere rianimazione cardiopolmonare [44], con un insieme specifico di norme.

In Italia non esiste una normativa specifica per l'ordine di non rianimare (*DNR*). Tuttavia, le disposizioni sulla rianimazione e le decisioni relative al fine vita sono trattate attraverso un quadro legale più ampio basato su consenso informato, DAT e *ACP* (PCC) [7].

**Fine vita**

La definizione di fine vita varia nella letteratura. Una definizione comune è quella che attribuisce un lasso di tempo alla lunghezza di vita stimata. A questo proposito, il periodo di tempo più comunemente citato è inferiore ai sei mesi di vita stimata [45]. Tuttavia, altra letteratura si concentra sugli ultimi giorni o ore di vita. Una chiara definizione dei lassi di tempo relativi al fine vita può supportare le decisioni condivise e la PCC, nonché la definizione delle politiche e dei programmi sanitari esistenti e futuri. A questo proposito, è stato proposto che il “fine vita” dovrebbe riferirsi ai pazienti che probabilmente moriranno entro 12 mesi, mentre il termine "morente" dovrebbe riferirsi ai pazienti negli ultimi giorni o ore di vita [46].

**Eutanasia**

L'atto di procurare intenzionalmente la morte immediata di un individuo competente e informato che lo ha richiesto liberamente, da parte di un professionista sanitario, mediante la somministrazione di sostanze [43].

In alcuni paesi del mondo, l'eutanasia è legale [24]; il primo Paese a legalizzarla è stato l’Olanda, il 1° aprile 2002. In Italia, l'eutanasia è un reato punibile ai sensi dell'articolo 579 del Codice Penale (omicidio del consenziente) [47].

Il termine "eutanasia passiva" è stato spesso utilizzato e persiste in alcuni contesti per descrivere decisioni come la sospensione del trattamento, spesso in modo insidiosamente ingannevole. Questa espressione è fuorviante e scorretta, poiché l'eutanasia è un atto attivo e volontario. L'espressione di questa volontarietà non è necessariamente attuale, ma può essere anticipata tramite una DA, come nel caso della legge olandese [24]. Se il medico prescrive e/o prepara il principio attivo in grado di causare la morte, principio attivo che il paziente si auto-somministra, si configura il SMA.

Allo stesso modo, l'omissione di un atto che porta alla morte non costituisce "eutanasia passiva" e può essere ricondotta solo a: (a) astensione da parte del medico, clinicamente fondata e giustificata, da qualsiasi irragionevole ostinazione nella somministrazione del trattamento e dall'uso di trattamenti inutili o sproporzionati (articolo 2 della Legge 219/2017) [7]; (b) rifiuto del paziente (attuale, anticipato da DA o ricostruito) (articoli 1, 4, 5 della Legge 219/2017) [7]. Vedi anche la voce “decisioni di non trattamento”.

**Trattamento di sostegno vitale (TSV)**

Una delle definizioni più autorevoli di TSV proviene dall'*American Medical Association* (*AMA*): “TSV è qualsiasi trattamento che serve a prolungare la vita senza risolvere la condizione medica sottostante. TSV può includere, ma non è limitato a, ventilazione meccanica, dialisi renale, chemioterapia, antibiotici, nutrizione e idratazione artificiali” [48].

TSV è uno dei quattro criteri obbligatori per legittimare il SMA nella Sentenza della CC 242/2019 [8]. Questo criterio è unico per l'Italia, poiché non è presente nei paesi dove il SMA è legalizzato o depenalizzato [24].

L'espressione "ma non è limitato a", nella suddetta definizione dell’*AMA*, evidenzia la complessità del criterio TSV, come sottolineato dal Comitato Nazionale di Bioetica nel contesto italiano [49]. Ad esempio, se un paziente tetraplegico ha una dipendenza assoluta e completa da un caregiver per il soddisfacimento delle proprie necessità vitali, come l'alimentazione, questo è considerato un TSV? Questo è stato riconosciuto come TSV nel 'Caso Anna' [50].

Inoltre, la CC è stata interpellata da un giudice referente per ampliare i criteri di accesso al SMA. Nella Sentenza 135/2024 [9], la CC ha meglio definito il significato di TSV, estendendolo ben oltre la sentenza precedente, per includere procedure - come l'evacuazione manuale delle feci, la cateterizzazione della vescica o la tosse assistita - solitamente eseguite da professionisti sanitari, ma che possono anche essere apprese dai familiari o dai *caregiver*, a condizione che la loro sospensione determini prevedibilmente la morte del paziente in breve tempo. La CC ha anche chiarito che, ai fini dell'accesso al SMA, non si può fare distinzione tra la condizione di un paziente già sottoposto a TSV, la cui interruzione il paziente può richiedere, e quella di un paziente non ancora sottoposto a tali trattamenti ma ora in bisogno di essi. Anche il paziente in quest'ultima condizione può legittimamente rifiutare di iniziare TSV, in base a quanto delineato nella Sentenza 242/2019 della CC [8, 9].

***Living will***

Un documento legale che fa parte delle DA contenente le volontà della persona riguardo ai trattamenti medici. Questa definizione viene anche usata (in modo che riteniamo improprio) come sinonimo di DA. In Italia i termini “testamento biologico” o “biotestamento” sono utilizzati nei media ma dovrebbero essere scoraggiati (vedi tabella).

**Decisioni di non trattamento**

L'atto di astenersi (non iniziare o intensificare) o sospendere (interrompere) un trattamento medico sia per motivi di futilità medica, sia su richiesta volontaria e competente della persona, anche come documentato nelle DA.

La sospensione o l’astensione dal trattamento non è quindi paragonabile all’eutanasia.

In Italia, la Legge 219/2017 [7] stabilisce che "nessun trattamento sanitario può essere iniziato o proseguito se privo del consenso libero e informato della persona interessata, tranne che nei casi espressamente previsti dalla legge" e riconosce il diritto di qualsiasi persona competente di rifiutare o interrompere qualsiasi trattamento, anche se essenziale per la propria sopravvivenza, comprese nutrizione e idratazione artificiali. Infatti, “il medico è tenuto a rispettare la volontà espressa dal paziente di rifiutare il trattamento medico o di rinunciare al medesimo e, in conseguenza di ciò, è esente da responsabilità civile o penale" (articolo 1 comma 6). La richiesta di sospensione del trattamento medico può essere associata alla richiesta di CP (inclusa la sedazione palliativa profonda continua) per alleviare la sofferenza del paziente.

**Cure Palliative (CP)**

La legge italiana 38/2010 definisce le CP come "l'insieme degli interventi terapeutici, diagnostici e assistenziali, rivolti sia alla persona malata sia al suo nucleo familiare, finalizzati alla cura attiva e totale dei pazienti la cui malattia di base, caratterizzata da un’inarrestabile evoluzione e da una prognosi infausta, non risponde più a trattamenti specifici" [51]. Questa definizione sottolinea che le CP dovrebbero essere offerte a tutti i pazienti che presentano sintomi e bisogni complessi, sia durante i trattamenti volti a rallentare la malattia (CP precoci e simultanee), sia quando tali trattamenti non sono più efficaci (CP di fine vita). Le CP mirano a prevenire o controllare/alleviare le sofferenze fisiche, psicologiche, sociali e spirituali (vedi la voce “dolore totale”) causate dalla condizione di base. Inoltre, si prefiggono di promuovere l'autodeterminazione del paziente, l'inclusione sociale e la comunicazione con i propri cari, assicurando la continuità delle cure in ogni contesto (dal domicilio all'ospedale), per migliorare la QdV del paziente, ponendo attenzione anche ai suoi familiari, fino alla fine della vita e, per i familiari, anche durante il lutto [52]. L'integrazione delle CP nelle unità di terapia intensiva ha dimostrato di ridurre la durata delle degenze ospedaliere, aumentare l'adozione di DA e diminuire l'uso di trattamenti di prolungamento della vita privi di beneficio [2, 53].

I benefici delle CP nel miglioramento degli esiti per i pazienti sono sempre più evidenti, in particolare per quanto riguarda il controllo dei sintomi, la QdV complessiva e la soddisfazione delle cure sia per i pazienti che per le famiglie, incluse le CP erogate ai pazienti neurologici [21-23]. Tuttavia, l'integrazione precoce delle CP nel percorso di malattia rimane una sfida. Inoltre, spesso manca chiarezza sulle definizioni usate e su chi sia responsabile dell’erogazione di tali servizi.

Esistono diversi modelli di erogazione delle CP. Un modello che intendiamo promuovere è l'approccio dinamico alle CP precoci e simultanee, che integra le CP fornite dai neurologi (“CP generali” o “CP primarie”) con le “CP specialistiche”. Queste ultime richiedono un team interprofessionale e interdisciplinare in grado di rispondere ai bisogni mutevoli del paziente e dei suoi cari [21, 54].

In un rapporto del 1996, il Comitato Etico e di Scienze Umane dell'*American Academy of Neurology* (*AAN*) dichiarò che fornire CP primarie è responsabilità di tutti i neurologi, posizione confermata nel *position paper* del 2022 [55, 56].

Dalla pubblicazione del rapporto del 1996, l'*AAN* ha sviluppato un vero e proprio campo della neuro-palliazione, configurandolo come una sotto-specialità della neurologia e delle CP e definendolo come un "approccio alle CP che si concentra sui bisogni specifici dei pazienti con malattie neurologiche e delle loro famiglie" [57]. Analogamente, l’*EAN* ha istituito un *Panel* Scientifico sulle CP e promosso un documento di consenso [21].

Le CP sono concettualmente contrarie sia all'anticipazione che al prolungamento della fase terminale della vita. Infatti, quasi tutte le definizioni di CP nel corso degli anni hanno confermato l'affermazione che "le CP considerano la morte un evento naturale e non accelerano né ritardano la morte" [58]. Anche nei Paesi in cui eutanasia e/o suicidio assistito sono legali (es. Paesi Bassi, Belgio, Lussemburgo, Canada) [24], il dibattito nel campo delle CP è sempre stato molto attivo, e le società scientifiche di CP hanno espresso opposizione a tali pratiche, sebbene vi sia una posizione minoritaria tra i professionisti sanitari che invece si è mostrata favorevole [59].

**Sedazione Palliativa**

“L'uso monitorato di farmaci destinati a indurre uno stato di coscienza ridotta o assente (incoscienza) per alleviare il peso di una sofferenza altrimenti intrattabile in modo eticamente accettabile per il paziente, la famiglia e il personale sanitario” [60].

Non esistono dati solidi basati su prove di efficacia che la sedazione palliativa acceleri la morte; in alcuni casi può, al contrario, prolungare brevemente la vita residua del paziente anche nel contesto di una malattia terminale imminente [61].

Conformemente alle definizioni suggerite dal Comitato Nazionale per la Bioetica [42], la sedazione palliativa può essere somministrata in diversi modi. In base al livello di coscienza, può essere moderata/superficiale (la coscienza non è completamente abolita) o profonda (la coscienza è completamente abolita). In base alla durata, può essere: temporanea (per un periodo limitato); intermittente (somministrata in modo alternato in base alle circostanze mutevoli); continua (prolungata fino alla morte del paziente, ossia sedazione palliativa continua profonda)."

**Suicidio medicalmente assistito (SMA)**

"Un medico che intenzionalmente aiuta una persona a terminare la propria vita fornendo farmaci per l'autosomministrazione, su richiesta volontaria e competente di quella persona" [43].

In Italia, il suicidio assistito è un reato punibile con la reclusione da cinque a dodici anni, secondo l'articolo 580 del Codice Penale (istigazione o aiuto al suicidio) [47].

La Sentenza n. 242/2019 della CC [8] ha introdotto un'eccezione alla punibilità del suicidio assistito. Infatti, la Corte ha dichiarato l’illegittimità dell’articolo 580 "nella parte in cui non esclude la punibilità di chi (...) agevola l’esecuzione del proposito di suicidio, autonomamente e liberamente formatosi, di una persona tenuta in vita da trattamenti di sostegno vitale e affetta da una patologia irreversibile, fonte di sofferenze fisiche o psicologiche che ella reputa intollerabili, ma pienamente capace di prendere decisioni libere e consapevoli, sempre che tali condizioni e le modalità di esecuzione siano state verificate da una struttura pubblica del servizio sanitario nazionale, previo parere del comitato etico territorialmente competente” [8]. Perché la sentenza sia applicabile, è necessario che la volontà della persona sia stata chiaramente ed inequivocabilmente espressa, compatibilmente con le sue condizioni. È inoltre richiesto che la persona sia stata adeguatamente informata sul SMA e sulle possibili soluzioni alternative, in particolare l’accesso alle CP e, se opportuno, alla sedazione palliativa.

In assenza di una normativa specifica, questa sentenza rappresenta la base per l’accesso attuale al SMA, che rimane lontano dall'essere pienamente chiarito, con procedure ancora disomogenee sul territorio nazionale. Per i requisiti stabiliti dalla CC, vedere anche le voci “capacità decisionale”, “trattamenti di sostegno vitale” e “prognosi sfavorevole”.

***Physician Orders for Life-Sustaining Treatment* (*POLST*)**

Tipo di DA destinato a persone a cui è stata diagnosticata una malattia grave. Si tratta di un documento vincolante, compilato dal medico curante del paziente, che specifica i trattamenti di sostegno vitale che devono essere somministrati al paziente in caso di incapacità decisionale [62]. In Italia, il concetto di POLST non è formalmente riconosciuto.

**Qualità di Vita (QdV)**

“La percezione che gli individui hanno della loro posizione nella vita nel contesto della cultura e dei sistemi di valori in cui vivono e in relazione ai loro obiettivi, aspettative, standard e preoccupazioni” [63].

La QdV è un concetto multidimensionale: un minimo di tre dimensioni interconnesse (fisica, psicologica e sociale) contribuiscono alla QdV complessiva dell'individuo [64]. Oltre alla multidimensionalità, la soggettività è una caratteristica fondamentale della QdV. Il miglior valutatore di questo costrutto è l'individuo stesso, utilizzando strumenti di autovalutazione. In ambito di ricerca clinica, l’impiego di strumenti standardizzati per la misura della QdV è aumentato esponenzialmente nel tempo, e sono stati prodotti e validati strumenti di QdV sia generici che specifici per varie condizioni, in diverse lingue e culture. In neurologia, la valutazione della QdV è cruciale per comprendere appieno l’impatto delle malattie e dei loro possibili trattamenti sulla vita dei pazienti [65]. Questo è valido anche per le CP, dove la QdV delle persone significative per il paziente è anch'essa un risultato chiave [52].

**Sintomo Refrattario**

“Un sintomo per il quale tutti i possibili trattamenti hanno fallito, o si stima che non siano disponibili metodi per la palliazione entro il lasso di tempo e il rapporto rischio–beneficio che il paziente può tollerare […] che non compromettano la coscienza” [60].

In altre parole, i sintomi refrattari sono presenti in situazioni in cui la migliore gestione possibile con trattamenti convenzionali ha fallito o non può essere utilizzata, come quando gli effetti collaterali sono inaccettabili per il paziente o gli effetti richiedono troppo tempo per alleviare il sintomo entro un intervallo di tempo appropriato [66].

I pazienti con grave disabilità neurologica spesso sperimentano una gamma di sintomi persistenti e angoscianti. Un elenco incompleto di sintomi che possono diventare resistenti alle terapie standard e sono classificati come refrattari include, ma non si limita a, dispnea, dolore, delirium e agitazione, nausea, crisi epilettiche e angoscia esistenziale (inclusa l'angoscia legata alla morte). Questi sintomi non si limitano a quelli fisici; pertanto, è necessario un approccio multidimensionale per valutare e gestire completamente la condizione del paziente [67].

Quando gli approcci terapeutici hanno fallito e i sintomi sono considerati refrattari al trattamento, in particolare nel fine vita, la sedazione palliativa diventa una scelta rilevante [60].

**Processo Decisionale Condiviso (*Shared Decision-Making - SDM*)**

Un modello basato su evidenze e principi etici nel quale decisioni informate vengono prese collaborativamente tra medici e pazienti, sulla base delle migliori evidenze disponibili e dei valori e preferenze del paziente [68-70]. La sua implementazione è particolarmente rilevante nel contesto di decisioni mediche “sensibili” alle preferenze, tipicamente in condizioni croniche caratterizzate da prognosi variabile e dalla limitata efficacia dei trattamenti modificanti la malattia. Questo è il caso della maggior parte delle malattie neurologiche, dove tali decisioni vengono prese dalla fase diagnostica alla fase finale della vita [71]. SDM e PCC accrescono l'autonomia del paziente coinvolgendo gli stessi, medici e, quando appropriato, le persone significative per il paziente nel processo decisionale. In molti Paesi la SDM è parte dei modelli di *best practice* e della formazione medica [72].

**Decisore Sostituto (Fiduciario)**

Una persona nominata per garantire il rispetto delle scelte del paziente nel caso in cui quest'ultimo non sia più in grado di farlo, sia in modo permanente che temporaneo. Non è obbligatorio identificare un decisore sostituto e, nella maggior parte dei Paesi occidentali, esiste un documento di DA specifico per questo scopo.

Secondo la Legge Italiana 219/2017 [7], spetta alla persona nominare o meno un decisore sostituto, sia per le DAT sia per la PCC. È importante notare che la Legge 219/2017 utilizza il termine specifico di “fiduciario” per indicare il decisore sostituto nelle DAT e nella PCC. In entrambe le procedure, possono essere nominati e elencati più fiduciari in ordine di preferenza, ognuno dei quali deve firmare il documento e fornire le proprie informazioni di contatto.

Per quanto riguarda la trasmissione di informazioni e il consenso informato (articolo 1, Legge 219/17) [7], il decisore sostituto è identificabile come incaricato (vedi Tabella). In particolare, il paziente "può rifiutare in tutto o in parte di ricevere le informazioni ovvero indicare i familiari o una persona di sua fiducia incaricati di riceverle e di esprimere il consenso in sua vece […]. Il rifiuto o la rinuncia alle informazioni e l’eventuale indicazione di un incaricato sono registrati nella cartella clinica e nel fascicolo sanitario elettronico” [7].

**Dolore totale**

Questo termine è stato introdotto da Cicely Saunders, la fondatrice del moderno movimento *hospice*, e comprende le dimensioni fisiche, emotive, sociali e spirituali della sofferenza [73].

**Prognosi infausta**

In assenza di una descrizione di questa espressione nel contesto della Legge 38/2010 [51], la prognosi infausta si riferisce a condizioni mediche in cui la possibilità di recupero è assente o estremamente limitata, accompagnata da un significativo declino della QdV del paziente e/o da una ridotta aspettativa di vita. Pertanto, questa definizione non dovrebbe limitarsi alle malattie che portano a morte, ma dovrebbe estendersi a situazioni in cui la condizione clinica ha un severo impatto esistenziale, richiedendo una gestione complessa dei sintomi che influiscono sulla QdV del paziente.

**Morte Volontaria Assistita (MVA)**

È definita come la scelta attiva e intenzionale di una persona di porre fine alla propria vita nel contesto di una malattia terminale e/o di una grave sofferenza. Questo termine comprende sia il SMA che l'eutanasia.

Le richieste di MVA da parte di pazienti con malattie neurologiche possono derivare da una complessa combinazione di fattori psicologici, fisici (ad esempio, dolore, affaticamento), sociali e spirituali. Queste richieste riflettono spesso una sofferenza complessiva e una perdita di autonomia e dignità a causa di un'incapacità di vivere secondo i valori e le preferenze individuali [74]. I pazienti in fase avanzata di malattia possono avvertire una perdita di controllo sulla propria vita [75], percependo la propria situazione come intollerabile e sperimentando un senso di perdita di dignità. Possono sentirsi un peso, sia finanziariamente che emotivamente, per le proprie famiglie, e l'isolamento sociale può intensificare il desiderio di morire. La depressione clinica, comune tra i pazienti con malattie terminali, può anche influenzare il desiderio di MVA (vedi “capacità decisionale”).

Linee guida dell*'EAN* suggeriscono di incoraggiare i pazienti a discutere i propri orientamenti riguardo alla MVA [22, 23, 76]. È anche importante notare che le CP, anche quando praticate secondo gli standard migliori, non possono prevenire i pazienti dal richiedere la MVA [43].

**BIBLIOGRAFIA**

1. Sallnow L, Smith R, Ahmedzai SH, et al (2022) Report of the Lancet commission on the value of death: Bringing death back into life. Lancet 399(10327):837–84.
2. Sprung CL, Ricou B, Hartog CS, et al (2019) Changes in end-of-life practices in European intensive care units from 1999 to 2016. JAMA;322(17):1692–704.
3. Welby P (2006) Lasciatemi morire. Rizzoli, Milano.

Viceconte N (2018) L’ultima tessera del domino: il “caso Englaro” è chiuso. A margine della sentenza del Consiglio di Stato n. 3058 del 2017). Corti supreme e salute 1:77-94.

AAVV (2020) La Corte costituzionale e il fine vita. Un confronto interdisciplinare sul caso Cappato-Antoniani. Ed. [D’Alessandro Giovanni](about:blank), [Di Giovine Ombretta](about:blank). Collana: [Law and Legal Institutions](about:blank). ISBN 9788892133679.

Chieffi L (2024) Suicidio assistito in Italia tra aperture giurisprudenziali e persistenti impedimenti nelle concrete prassi. Rivista Associazione Italiana Costituzionalisti 1. [https://www.rivistaaic.it/it/rivista/ultimi-contributi-pubblicati/lorenzo-chieffi/suicidio-assistito-in-italia-tra-aperture-giurisprudenziali-e-persistenti-impedimenti-nelle-concrete-prassi](about:blank). Accessed 5 August 2024.

Repubblica Italiana (2018) Legge 219/2017 “Norme in materia di consenso informato e di disposizioni anticipate di trattamento”. Gazzetta Ufficiale della Repubblica Italiana S.G. n. 12, 16 January 2018. https://www.gazzettaufficiale.it/eli/id/2018/1/16/18G00 006/sg. Accessed 5 August 2024.

Italian Constitutional Court (2019) Judgment 242/2019. [https://www.cortecostituzionale.it/documenti/download/doc/recent_judgments/Sentenza_n_242_del_2019_Modugno_en.pdf](about:blank) Accessed 7 August 2024.

1. Italian Constitutional Court (2024) Judgment 135/2024. https://www.cortecostituzionale.it/documenti/comunicatistampa/CC_CS_20240718154806.pdf. Accessed 2 September, 2024.
2. Provinciali L, Carlini G, Tarquini D, et al (2016) Need for palliative care for neurological diseases. Neurol Sci. 37(10): 1581-7.
3. Bombaci A, Di Lorenzo F, Pucci E, Solari A, Veronese S; Società Italiana di Neurologia–Società Italiana di Cure Palliative Intersociety Table (2024). Eur J Neurol. 31(9):e16376.
4. Ugolino F (2009) Il caso di Eluana Englaro e le vicende politico-istituzionali. In: Civitas Europa 22: 155-8.
5. Pontificia Accademia per la Vita (2024) Piccolo lessico del fine-vita. Collana Humana Communitas. Amministrazione del Patrimonio della Sede Apostolica. Dicastero per la Comunicazione – Libreria Editrice Vaticana. Città del Vaticano. ISBN 978-88-266-0895-2.
6. Senato Repubblica Italiana (S. n. 1083, XIX legislatura, “Modifiche all'articolo 580 del Codice Penale e modifiche alla Legge 22 dicembre 2017, n. 219, in materia di Disposizioni Anticipate di Trattamento e prestazione delle Cure Palliative”. Comunicato alla Presidenza - 26 Marzo 2024. https://www.senato.it/japp/bgt/showdoc/REST/v1/showdoc/get/fragment/19/DDLPRES/0/1411702/all. Accessed 3 September 2024.
7. National Hospice and Palliative Care Ethics Committee (2010) Commentary and position statement on artificial nutrition and hydration. Approved by the National Hospice and Palliative Care Organization (NHPCO) Board of Directors on September 12, 2010. https://www.nhpco.org/wpcontent/uploads/2019/04/ANH_Statement_Commentary.pdf. Accessed: 3 August 2024.

American Academy of Hospice and Palliative Medicine (AAHPM) Board of Directors (2013) Statement on Artificial Nutrition and Hydration Near the End of Life. September 13, 2013. [https://aahpm.org/positions/anh](about:blank) Accessed 3 August 2024.

1. Druml C, Ballmer PE, Druml W, et al (2016) ESPEN guideline on ethical aspects of artificial nutrition and hydration. Clin Nutr 35(3):545-56.
2. Congedo M, Causarano RI, Alberti F, et al (2010) Bioethics and Palliative Care in Neurology Study Group of Italian Society of Neurology. Ethical issues in end of life treatments for patients with dementia. Eur J Neurol 17(6):774-9.
3. Tarquini D, Congedo M, Formaglio F, et al. (2012) Persistent vegetative state: an ethical reappraisal. Neurol Sci. 33(3):695-700.
4. Tarquini D, Pucci E, Gasparini M, et al; Gruppo di Studio in Bioetica e Cure Palliative della Società Italiana di Neurologia (2014) Diagnosing Alzheimer's disease: from research to clinical practice and ethics. Recenti Prog Med 105(7-8):295-9.
5. Oliver DJ, Borasio GD, Caraceni A, et al (2016) A consensus review on the development of palliative care for patients with chronic and progressive neurological disease. Eur J Neurol 23:30–8.
6. Solari A, Giordano A, Sastre-Garriga J, et al (2020) EAN guideline on palliative care of people with severe, progressive Multiple sclerosis. Eur J Neurol 27(8):1510-29.
7. Van Damme P, Al-Chalabi A, Andersen PM, et al. (2024) European Academy of Neurology (EAN) guideline on the management of amyotrophic lateral sclerosis in collaboration with European Reference Network for Neuromuscular Diseases (ERN EURO- NMD). Eur J Neurol 31: e16264.
8. Pucci E, Ticozzi N, Comi G, Mancardi G, Provinciali L, Padovani A, Solari A (submitted) Neurology and Physician-Assisted Suicide: Position of the Italian Society of Neurology, SIN.
9. Sudore RL, Lum HD, You JJ, et al (2017) Defining Advance Care Planning for Adults: A Consensus Definition from a Multidisciplinary Delphi Panel. J Pain Symptom Manage 53(5): 821-832.e1.
10. House SA, Schoo C, Ogilvie WA (2023) Advance Directives. [Updated 2023 Aug 8]. In: StatPearls [Internet]. Treasure Island (FL): StatPearls Publishing; 2024 Jan-. Available from: [https://www.ncbi.nlm.nih.gov/books/NBK459133/](about:blank)
11. Costituzione della Repubblica Italiana (1948) Gazzetta Ufficiale, 27 dicembre 1947, n. 298.
12. Russell JA, Epstein LG, Bonnie RJ et al (2018) On behalf of the Ethics, Law, and Humanities Committee (a joint committee of the AAN, ANA, and CNS). Lawful physician-hastened death. AAN position statement. Neurology 90: 420-22.
13. World Health Organization (2016) WHO definition of palliative care. Geneva: World Health Organization.

Goni-Fuste B, Crespo I, Monforte- Royo C, et al (2021) What defines the comprehensive assessment of needs in palliative care? An integrative systematic review. Palliat Med 35:651–69.

1. Snowden A, Young J, Roberge D, et al (2023) Holistic needs assessment in outpatient cancer care: a randomised controlled trial. BMJ Open 13: e066829.
2. American Psychiatric Association (1998) Guidelines for assessing the decision-making capacities of potential research subjects with cognitive impairment. Am J Psychiatry 155(11):1649-50.
3. Appelbaum PS (2007) Assessment of patient’s competence to consent to treatment. N Engl J Med 357(18): 1834-40.
4. Barstow C, Shahan B, Roberts M. (2018) Evaluating medical decision-making capacity in practice. Am Fam Physician 98(1): 40-6.
5. Gruppo di lavoro in materia di aiuto medico a morire (2019) Aiuto medico a morire e diritto: per la costruzione di un dibattito pubblico, plurale e consapevole. Recenti Prog Med 110(10):462-472. doi 10.1701/3246.32161.

Breitbart W, Rosenfeld B, Pessin H, et al (2000) Depression, hopelessness, and desire for hastened death in terminally ill patients with cancer. JAMA 284(22): 2907-11.

Emanuel EJ, Fairclough DL, Emanuel LL, et al (2000) Attitudes and Desires Related to Euthanasia and Physician-Assisted Suicide Among Terminally Ill Patients and Their Caregivers. JAMA 284(19):2460-8.

1. Tecuta L, Tomba E, Grandi S, Fava GA (2015) Demoralization: a systematic review on its clinical characterization. Psychol Med. 45(4):673-91.
2. Dierickx S, Deliens L, Cohen J, Chambaere K (2017) Euthanasia for people with psychiatric disorders or dementia in Belgium: analysis of officially reported cases. BMC Psychiatry 17(1): 203.

van den Bosch A, Marijnissen RM, Hanssen DJC, Oude Voshaar RC (2024) Capacity assessment for euthanasia in dementia: A qualitative study of 60 Dutch cases. J Am Geriatr Soc 1‐11. doi:10.1111/jgs.19218.

Scopetti M, Moreno D, Padovano M, et al (2023) Assisted suicide and euthanasia in mental disorders: Ethical positions in the debate between proportionality, dignity, and the right to die. Healthcare 11:1470.

1. Comitato Nazionale per la Bioetica (2016) Sedazione palliativa profonda continua nell’imminenza della morte. <https://bioetica.governo.it/media/1804/p122_2016_sedazione_profonda_it.pdf> Accessed 14 October 2024.
2. Radbruch L, Leget C, Bahr P, et al (2016) Euthanasia and physician-assisted suicide: A white paper from the European Association for Palliative Care. Palliat Med 30(2):104-16.
3. Vranick J, Sanghavi DK, Torp KD, et al (2022) Do not resuscitate. [Updated 2022 Sep 26]. In: StatPearls [Internet]. Treasure Island (FL): StatPearls Publishing; 2024 Jan-. Available from: [https://www.ncbi.nlm.nih.gov/books/NBK470163/.](about:blank)
4. Huffman JL, Harmer B (2023) End-of-Life Care. [Updated 2023 Feb 20]. In: StatPearls [Internet]. Treasure Island (FL): StatPearls Publishing; 2024 Jan–.PMID: 31334996 Free Books & Documents.
5. Leadership Alliance for the Care of Dying People (2014) One Chance to Get It Right. Publications Gateway Reference 01509. <https://assets.publishing.service.gov.uk/media/5a7e301ced915d74e33f09ee/One_chance_to_get_it_right.pdf> Accessed: 14 October 2024.

Codice penale. Regio Decreto 19 ottobre 1930, n. 1398 aggiornato alla L. n. 114/2024. [https://www.altalex.com/documents/codici-altalex/2014/10/30/codice-penale.](about:blank) Accessed 7 September 2024.

1. Council on Ethical and Judicial Affairs, American Medical Association (1992) Decisions near the End of Life. JAMA 267(16): 2229-33.

Comitato Nazionale per la Bioetica (2024) Risposta. Quesito del Comitato Etico Territoriale della Regione Umbria. 3 Novembre 2023. https://bioetica.governo.it/media/titp0sf3/risposta-tsv-rev-2-luglio-2024-finale.pdf. Accessed 14 October 2024.

AAVV. Relazione della commissione medica multidisciplinare dell’Azienda Sanitaria Universitaria Giuliano Isontina. (03.08.23) https://associazionelucacoscioni.it/wp-content/uploads/2023/08/doc02725820230807113736.pdf?_gl=1*jhuuqw*_up*MQ..*_ga*NDIzMzA3MjA0LjE3MjAzODgyNDg.*_ga_QQMZLR1HRV*MTcyMDM4ODI0Ny4xLjAuMTcyMDM4ODI0Ny4wLjAuMA Accessed 8 July 2024.

Repubblica Italiana (2010). Legge 38/2010 “Disposizioni per garantire l'accesso alle cure palliative e alla terapia del dolore”. Gazzetta Ufficiale Serie Generale n.65 del 19-03-2010 (10G0056). [https://www.gazzettaufficiale.it/gunewsletter/dettaglio.jsp?service=1&datagu=2010-03-19&task=dettaglio&numgu=65&redaz=010G0056&tmstp=1269600292070](about:blank) Accessed 8 July 2024.

1. Sepulveda C,Marlin A, YoshidaT,Ullrich A (2002) Palliative care: the World Health Organization’s global perspective. J Pain Sympt Manage 24: 91-6.
2. Braus N, Campbell TC, Kwekkeboom KL, et al (2016) Prospective study of a proactive palliative care rounding intervention in a medical ICU. Intensive Care Med 42(1): 54-62.

Oliver D (2018) Improving patient outcomes through palliative care integration in other specialised health services: what we have learned so far and how can we improve? Ann Palliat Med 7(3):S219-S230.

1. The American Academy of Neurology Ethics and Humanities Subcommittee (1996) Palliative care in neurology. Neurology 46: 870-2.

Taylor LP, Besbris JM, Graf WD, et al (2022) Clinical Guidance in Neuropalliative Care: An AAN Position Statement. Neurology 98:409-16.

1. Neuropalliative Care: A Guide to Improving the Lives of Patients and Families Affected by Neurological Disease (2019) Creutzfeldt CJ, Kluger BM, Holloway RG, Eds. Springer. ISBN: 978-3-31-993214-9.
2. Italian Society of Palliative Care (SICP), Position of the SICP on Euthanasia and Medically Assisted Suicide. Hearing of the SICP at the Italian Chamber of Deputies on Euthanasia and Medically Assisted Suicide, April 3, 2019. https://www.sicp.it/informazione/comunicati/2019/04/audizione-della-sicp-alla-camera-dei-deputati-su-eutanasia-e-suicidio-medicalmente-assistito/ Accessed 11 August 11 2024.
3. Gerson SM, Koksvik GH, Richards N, et al (2020) The Relationship of Palliative Care with Assisted Dying where Assisted Dying is Lawful: A Systematic Scoping Review of the Literature. J Pain Symptom Manage 59(6): 1287–303.e1.
4. Cherny NI, Radbruch L; Board of the European Association for Palliative Care (2009) European Association for Palliative Care (EAPC) recommended framework for the use of sedation in palliative care. Palliat Med 23:581–93.
5. Maltoni M, Scarpi E, Rosati M, et al (2012) Palliative sedation in end-of-life care and survival: A systematic review. J Clin Oncol 30:1378-83.
6. Figueroa Gray M, Randall S, Banegas M, et al (2024) Personal legacy and treatment choices for serious illness: a scoping review. BMJ Support Palliat Care. 2024 Jan 24: spcare-2023-004439. doi: 10.1136/spcare-2023-004439. Online ahead of print.
7. The WHOQOL Group (1995) The World Health Organization Quality of Life assessment (WHOQOL): Position paper from the World Health Organization. Soc Sci Med 41(10):1403-9.
8. European Medicines Agency (2005). Committee for medicinal products for human use (CHMP) reflection paper on the regulatory guidance for the use of health-related quality of life (HRQL) measures in the evaluation of medicinal products. [https://www.ema.europa.eu/en/documents/scientific-guideline/reflection-paper-regulatory-guidance-use-health-related-quality-life-hrql-measures-evaluation-medicinal-products_en.pdf](about:blank) Accessed 3 September, 2024.
9. Cella D, Nowinski C, Peterman A, et al (2011) The Neurology Quality of Life Measurement Initiative. Arch Phys Med Rehabil 92 (10): S28–S36.
10. Payne SA, Hasselaar J (2020) European Palliative Sedation Project. J Palliative Med 23(2): 154-5.
11. Klein C, Voss R, Ostgathe C, Schildmann JA; the SEDPALL study group (2023) Sedation in palliative care—a clinically oriented overview of guidelines and treatment recommendations. Dtsch Arztebl Int 120: 235–42.
12. Charles C, Gafni A, Whelan T (1999) Shared decision-making in the medical encounter: what does it mean? (or it takes at least two to tango). Soc Sci Med 44:681–92.
13. Barry MJ, Edgman-Levitan S (2012) Shared decision making–pinnacle of patient-centered care.N Engl J Med 366: 780–1.
14. Parsa-Parsi RW (2017) The revised declaration of Geneva. A modern-day physician‘s pledge. JAMA 318: 1971–2.
15. Heesen C, Solari A (2023) Shared decision-making in neurology. Front Neurol 14: 1222433. doi: 10.3389/fneur.2023.1222433.
16. CanMEDS (2015) Physician Competency Framework. Frank JR, Snell L, Sherbino J, Eds. Ottawa: Royal College of Physicians and Surgeons of Canada.
17. Richmond C (2005) Dame Cicely Saunders. BMJ 331(7510): 238.
18. Mavroudis I, Alexiou P, Petridis F, et al (2024) Patients' and caregivers' attitudes towards patient assisted suicide or euthanasia in amyotrophic lateral sclerosis-a meta-analysis. Acta Neurol Belg doi: 10.1007/s13760-024-02578-x. Online ahead of print.
19. Chochinov HM, Hack T, Hassard T, et al (2005) Understanding the will to live in patients nearing death. Psychosomatics 46(1): 7-10.
20. Kopke S, Giordano A, Veronese S et al (2019) Patient and caregiver involvement in the formulation of guideline questions: findings from the European Academy of Neurology guideline on palliative care of people with severe multiple sclerosis. Eur J Neurol 26(1):41-50. doi: 10.1111/ene.13760.
